# Recommendations To Prevent Taxonomic Misidentification of Genome-Sequenced Fungal Strains

**DOI:** 10.1128/MRA.01074-20

**Published:** 2021-12-02

**Authors:** Jos Houbraken, Cobus M. Visagie, Jens C. Frisvad

**Affiliations:** a Westerdijk Fungal Biodiversity Institute, Utrecht, The Netherlands; b Department of Biochemistry, Genetics, and Microbiology, Forestry and Agricultural Biotechnology Institute, University of Pretoria, Pretoria, South Africa; c Department of Biotechnology and Biomedicine, Technical University of Denmark, Kongens Lyngby, Denmark; Vanderbilt University

## LETTER

Correct identification of a (genome-sequenced) strain is an essential step in evolutionary and comparative genomic studies. It came to our attention that the number of publicly available misidentified genome-sequenced strains is increasing. By using the order *Eurotiales* (Aspergillus, *Penicillium*, *Talaromyces*, and related genera) as an example, in this letter we want to increase awareness among readers of *Microbiology Resource Announcements* of this ongoing problem and give recommendations to ensure availability and correct strain identification in the future.

**FIG 1:**
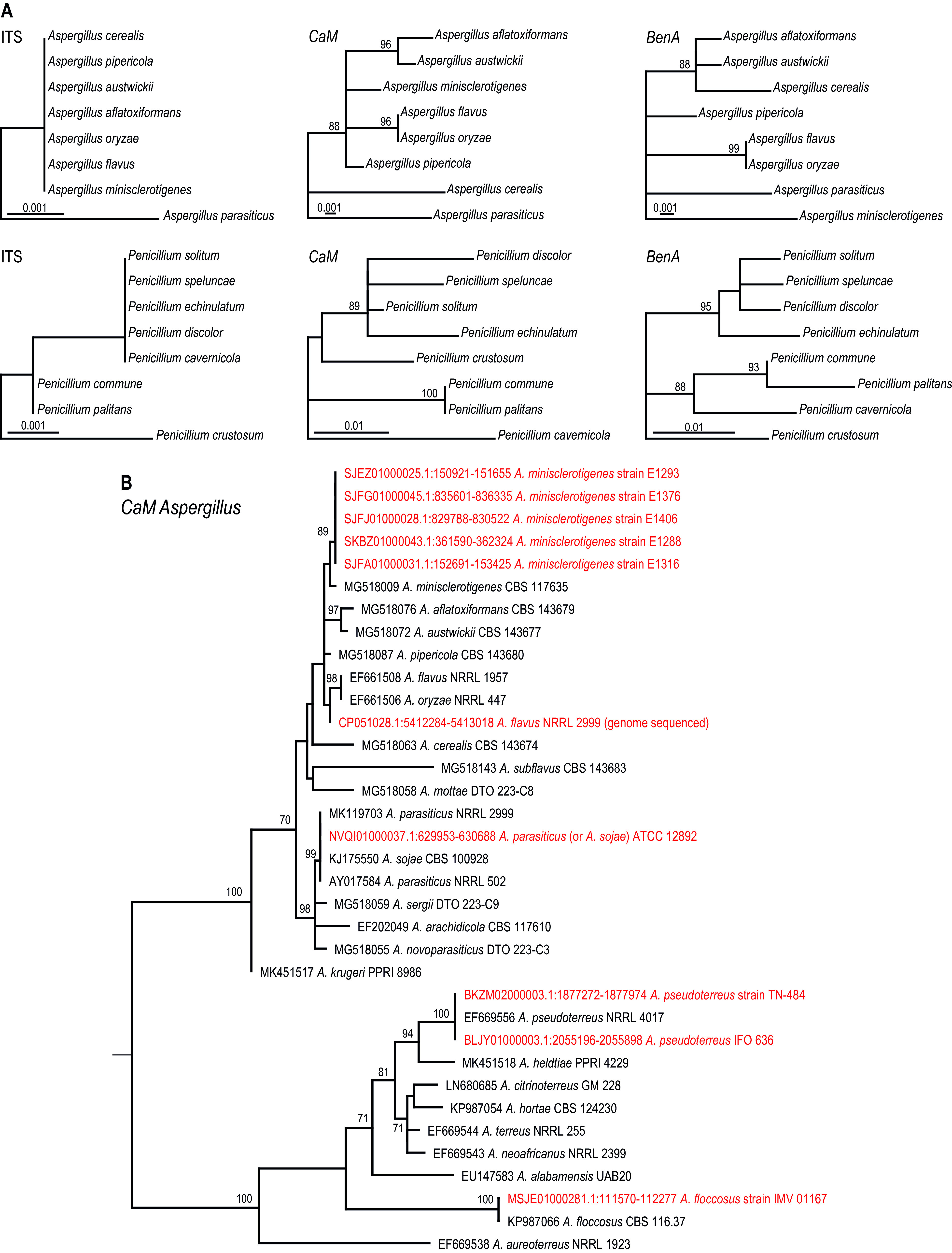
(A) Phylogenetic analysis showing the relationships of Aspergillus flavus and related species (top row) and Penicillium solitum and related species (bottom row). The phylogram based on the ITS barcode has low resolution, and greater variability is present in the *BenA* and *CaM* trees. (B) Phylogram based on *CaM* gene sequences of Aspergillus section *Flavi* and *Terrei* reference strains (9) and incorrectly identified genome-sequenced strains (indicated in red). The GenBank accession numbers are listed before the species name and strain numbers are listed afterward.

Species identification is an important step in biological research. A correct name is vital for optimal communication and is the link between studies in various fields. Currently, the identification of fungi relies mainly on (single) gene sequencing, and this approach has largely replaced identification methods using phenotypic and physiological characteristics. The internal transcribed spacer (ITS) region was accepted as the primary fungal barcode (1) and is recommended for the identification of uncharacterized fungal strains (without any *a priori* knowledge). Although the ITS is the most commonly used region, with a good track record for identifying species, there are many genera for which it does not provide enough resolution. Various studies showed that this locus cannot be used for identification of species in well-known genera such as Aspergillus, *Cladosporium*, Fusarium, *Penicillium*, and *Talaromyces* (2–6). In these genera, protein-coding genes are commonly used for identification and generally have higher interspecies variability than the ITS region. Unfortunately, there is no standard choice of a protein-coding gene for the identification of fungal isolates across different groups. Efforts have been made to assess potential candidate gene regions (and corresponding universal primer pairs) as secondary DNA barcodes (7). Translation elongation factor 1-α (*tef1*-α) is widely used as a phylogenetic marker in mycology and is used as a secondary identification barcode for various genera; however, standardization is lacking. While it has sufficient resolution in many genera (e.g., *Cladosporium* and Fusarium), *tef1*-α has never been extensively studied in Aspergillus, *Penicillium*, and related genera (order *Eurotiales*); therefore, databases (e.g., GenBank) lack reference sequences of this locus for these genera. With the exception of Aspergillus, partial β-tubulin (*BenA*) gene sequencing is recommended for *Penicillium*, *Talaromyces*, *Paecilomyces*, and related genera (8–10). Partial calmodulin (*CaM*) gene sequencing is recommended as an identification barcode for Aspergillus; however, *BenA* sequencing generally also works well. Both species markers perform better than ITS (8–11). Two examples are given in Fig. 1. The (ex-)type cultures of Aspergillus aflatoxiformans, Aspergillus austwickii, Aspergillus cerealis, Aspergillus flavus, Aspergillus minisclerotigenes, Aspergillus oryzae, and Aspergillus pipericola (9) have the same ITS sequence, while the majority have unique *BenA* and *CaM* sequences (with the exception of A. flavus and its domesticated form A. oryzae). Similarly, Penicillium cavernicola, Penicillium discolor, Penicillium echinulatum, Penicillium solitum, and Penicillium speluncae share the same ITS sequence but differ in their *BenA* and *CaM* gene sequences. In summary, ITS is the primary barcode but might lack resolution in some genera at the species level; in those cases, an additional marker is needed. There is no consensus regarding a secondary marker, and this needs to be determined for each genus. If needed, contact a taxonomist who can advise on the barcode(s) to use.

Correct identification also depends on the quality of the database. GenBank is generally used for strain identification, and users should be aware that sequences of incorrectly identified strains occur in GenBank (12, 13), leading to incorrect identifications. To date, there are no cutoff scores for species identification, and the variability differs according to marker and species (14). In the future, when more fungal genome sequences become available, average nucleotide identity (ANI) analyses could be applied to determine species boundaries and to confirm identifications, a method that is already used in bacteriology, where more genome sequences are available (15). In cases in which BLAST analysis results are not conclusive, it is recommended to construct a phylogenetic tree to determine the closest relatives of the strain. Lists of accepted Eurotialean species that include reference partial β-tubulin and calmodulin gene sequence data have been constructed and are a helpful aid for researchers to create phylogenetic trees based on reference sequence data and to obtain a correct identification (9). Similar lists have been prepared for other genera (16–18).

The number of genome sequencing projects has increased tremendously in recent years. It came to our attention that there is a continuing problem regarding incorrect identification and the unavailability of genome-sequenced strains. An overview of the genome-sequenced strains published in *Microbiology Resource Announcements* (including *Genome Announcements*) that belong to the order *Eurotiales* (Aspergillus, *Penicillium*, and related genera) was generated (19). Fifty-eight articles (from February 2013 to 31 March 2021), covering 141 Eurotialean strains, were published in *Microbiology Resource Announcements* and *Genome Announcements* (19). Of those strains, 18% (*n* = 26) were inadequately or wrongly identified (see Table 1 and the example of Aspergillus sections *Flavi* and *Terrei* in Fig. 1B), and 75% (*n* = 106) were not deposited in a public culture collection. These misidentifications can lead to incorrect conclusions. For example, the genome sequence of ATCC 48735, an environmental strain of Penicillium capsulatum (20), is actually that of Penicillium canescens. The genome data were later used in a comparative genomic analysis with a clinical P. capsulatum strain (21). Another example is the genome sequences of two P. solitum strains (22, 23) that are reported to be used for deepening the understanding of the genetic differences in, for example, mycotoxin production. Reidentification based on the available genome data showed that those P. solitum strains were actually Penicillium polonicum (RS1) and Penicillium crustosum (NJ1). More recently, the genomes of a set of 16 Aspergillus flavus and Aspergillus parasiticus strains were sequenced (24). These strains were selected based on the genetic fingerprints of 25 insertion/deletion markers within the aflatoxin biosynthesis pathway. Analyses of these markers will give insight into the potential of these strains to produce aflatoxin but are not recommended for species identification (11). Reidentification of the strains using the calmodulin barcode gene extracted from the genome sequence showed that 5 strains are actually A. minisclerotigenes (listed as A. flavus S-type, referring to the small-sized sclerotia the strain produces) (25). In 2008, Pildain et al. (26) showed that production of small-sized sclerotia is not a characteristic that can be attributed to one species but multiple A. flavus-like species (Aspergillus series *Flavi*), including A. minisclerotigenes, can produce these. This example illustrates that, besides a rigid sequence comparison, it is also important to use the most up-to-date taxonomic schemes and insights. The focus of our letter was the order *Eurotiales*, but similar issues may exist for other groups of fungi as well. For example, *Cladosporium* sp. strain TM138 (27) can be identified as Cladosporium halotolerans (based on partial *tef1* and actin gene sequences) and Aureobasidium pullulans var. *aubasidani* (28) as Aureobasidium pullulans (based on ITS and partial RNA polymerase II second largest subunit sequence data).

**TABLE 1:** Overview of inaccurately and inadequately genome-sequenced *Eurotiales* strains published in *Genome Announcements* and *Microbiology Resource Announcements* between February 2013 and 31 March 2021

Strain	Reported identity	Correct identity	Remarks	Reference
IFM 58123	Aspergillus awamori	Aspergillus welwitschiae	Incorrect identification	31
Strain E1288	Aspergillus flavus	Aspergillus minisclerotigenes	Incorrect identification	24
Strain E1293	Aspergillus flavus	Aspergillus minisclerotigenes	Incorrect identification	24
Strain E1316	Aspergillus flavus	Aspergillus minisclerotigenes	Incorrect identification	24
Strain E1376	Aspergillus flavus	Aspergillus minisclerotigenes	Incorrect identification	24
Strain E1406	Aspergillus flavus	Aspergillus minisclerotigenes	Incorrect identification	24
NRRL 5109	Aspergillus neoellipticus	Aspergillus fumigatus	Incorrect identification	32
Strain An76	Aspergillus niger	Aspergillus tubingensis	Incorrect identification	33
ATCC 12892	Aspergillus oryzae	Aspergillus parasiticus (or Aspergillus sojae)	Incorrect identification	34
NRRL 2999	Aspergillus parasiticus	Aspergillus flavus	Original strain differs from genome-sequenced strain	35
Strain TN-484	Aspergillus terreus	Aspergillus pseudoterreus	Incorrect identification	36
Strain IMV 01167	Aspergillus terreus	Aspergillus floccosus	Incorrect identification	37
IFO 6365	Aspergillus terreus	Aspergillus pseudoterreus	Incorrect identification	38
Strain BYSS01	Byssochlamys sp.	Monascus floridanus	Incorrect identification	39
Strain AF001	Byssochlamys sp.	Paecilomyces dactylethromorphus	Incorrect identification	40
Strain no. 5 (= NBRC 109023)	Byssochlamys spectabilis/Paecilomyces variotii	Paecilomyces formosus	Incorrect identification	41
Strain FENG	Paecilomyces hepiali	*Samsoniella* sp. (Cordycipitaceae)	Incorrect identification	42
ATCC 48735	Penicillium capsulatum	Penicillium canescens	Incorrect identification	20
Strain P2niaD18	Penicillium chrysogenum	Penicillium rubens	Incorrect identification	43
ATCC 18224	Penicillium marneffei	Talaromyces marneffei	Incorrect identification	44
Strain 113	Penicillium sclerotiorum	Penicillium maximae	Incorrect identification	45
Strain NJ1	Penicillium solitum	Penicillium crustosum	Incorrect identification	22
Strain RS1	Penicillium solitum	Penicillium polonicum	Incorrect identification	23
Strain SPG-F1	*Penicillium* sp.	Penicillium solitum	Inadequate identification	46
Strain SPG-F15	*Penicillium* sp.	Penicillium commune (or Penicillium camemberti, depending on colony morphology)	Inadequate identification	46
Strain Y-94 (= CBS 136886)	Talaromyces cellulolyticus	Talaromyces pinophilus	Incorrect identification	47

Finally, we would like to highlight that species and genus names can change due to new taxonomic insights. However, old names remain in the literature and, for scientists who are unaware of these taxonomic changes, literature with old taxonomic names might be overlooked or misinterpreted. For example, the genome-sequenced strain Trichoderma harzianum T6776 was correctly identified in 2015, but this strain is identified as Trichoderma afroharzianum using the current taxonomic classification (18). Similarly, Talaromyces marneffei was originally described in *Penicillium* (as Penicillium marneffei), and both names can occur in the literature (29).

Here, we want to increase awareness among scientists to use up-to-date taxonomic schemes in order to avoid incorrect identification and to ensure that a strain is available for the scientific community. We recommend the following steps before publication of genome sequences in the public domain. (i) Perform an identification using the latest taxonomic insights. If needed, contact a taxonomist who can advise regarding the current identity of the strain. (ii) Compare the identity of the original strain with the genome-sequenced strain. For example, NRRL 2999 was originally an Aspergillus parasiticus strain but is A. flavus based on the genome sequence (19, 30). In addition to the strain identification before genome sequencing, it is recommended to extract relevant gene regions from the genome obtained to confirm a correct identification. (iii) The strains should be deposited in at least one, but preferably two or more, recognized, public culture collections (from two countries). This would guarantee that the strain is (easily) accessible for other researchers and for future research purposes. (iv) If the project involves sequencing a representative of a species, make sure that the selected strain is typical of the species. In this case, it is important to study the phenotype of the strains. Type strains (and other [old] strains in culture collections) are not always the best choice, because these strains might have been preserved over a long time and could be deteriorated.

### Data availability.

The data that support the findings of this study are openly available in Figshare (https://doi.org/10.6084/m9.figshare.c.5360423.v1) (19).
